# Domestic water and sanitation as water security: monitoring, concepts and strategy

**DOI:** 10.1098/rsta.2012.0420

**Published:** 2013-11-13

**Authors:** David J. Bradley, Jamie K. Bartram

**Affiliations:** 1 Department of Zoology, Oxford University, Oxford OX1 3PS, UK; 2 Department of Disease Control, London School of Hygiene & Tropical Medicine, London WC1E 7HT, UK; 3 Department of Environmental Sciences and Engineering, Gillings School of Global Public Health, University of North Carolina at Chapel Hill, NC 27599, USA; 4 Water Institute, Gillings School of Global Public Health, University of North Carolina at Chapel Hill, NC 27606, USA

**Keywords:** water, sanitation, water security, millennium development goals, water monitoring, human right

## Abstract

Domestic water and sanitation provide examples of a situation where long-term, target-driven efforts have been launched with the objective of reducing the proportion of people who are water-insecure, most recently through the millennium development goals (MDGs) framework. Impacts of these efforts have been monitored by an increasingly evidence-based system, and plans for the next period of international policy, which are likely to aim at universal coverage with basic water and sanitation, are being currently developed. As distinct from many other domains to which the concept of water security is applied, domestic or personal water security requires a perspective that incorporates the reciprocal notions of provision and risk, as the current status of domestic water and sanitation security is dominated by deficiency This paper reviews the interaction of science and technology with policies, practice and monitoring, and explores how far domestic water can helpfully fit into the proposed concept of water security, how that is best defined, and how far the human right to water affects the situation. It is considered that they fit well together in terms both of practical planning of targets and indicators and as a conceptual framework to help development. The focus needs to be broad, to extend beyond households, to emphasize maintenance as well as construction and to increase equity of access. International and subnational monitoring need to interact, and monitoring results need to be meaningful to service providers as well as users.

## 1. Introduction: domestic water security

Provision of water for human domestic use can be viewed as a fundamental example of water security: survival is impossible without consuming water in some form, but sufficient water for survival alone is far from adequate for a tolerable or healthy life. Increasing volumes of water for diverse domestic uses benefits personal and family life, livelihood and human health [1–3]. Water quality will also influence particularly human health and disease prevention.

This review follows and contributes to the discussion on water security that began at a conference on that topic in Oxford in April 2012. Water security has been proposed as a possible leading concept for post-2015 sustainable development goals to follow the millennium development goals (MDGs) [4]. The water, sanitation and hygiene (WaSH) area is concerned with domestic water and sanitation, and associated behaviour, to derive benefit from them and cause no harm to others.

The increasing provision of water and sanitation (W&S) facilities for the world's inhabitants has, for the past quarter-century, taken place beneath the umbrella of the MDGs that have goals to inspire, and targets to give substance to lofty aims. W&S therefore already has a relatively well-developed structure of targets, indicators and metrics [5]; and during 2012 technical working groups convened by WHO and UNICEF worked towards devising possible interdependent targets and indicators for WaSH post-2015 [5]. There are many other global and local water issues beyond domestic WaSH, as discussed in Grey *et al.* [6], and many of these can fit comfortably within an overall theme, or goal, of water security. This review explores the question of whether WaSH activities and problems can also fit beneficially into the water security theme, not just as a particular political convenience if the situation arises, but in a deeper sense.

In the course of analysing this question, two levels of questioning have emerged. One concerns this as a practical question relating to targets and indicators in any goal-orientated future architecture involving water security. The other asks how far the concept of water security, plus that of the human right to water and sanitation [7], can provide a conceptual framework for formulating, studying and tackling the issues and problems confronting WaSH development in the coming period. They are similar but different questions. We answer both in the affirmative.

In applying a water security perspective to the problems of domestic water globally, we consider here the meaning of water security for water and sanitation. It is instructive to compare the two recent definitions of water security proposed by Grey. The most recent, is ‘water security is a tolerable level of water-related risk to society’ [6]. It reflects the sombre outlook for overall water security, of which water used for domestic water and sanitation is a small but important part. However, the complete emphasis on *risk* is most appropriate for populations who already have something and are looking at the consequences of it being taken away. By contrast, an earlier Grey & Sadoff [8] definition ‘the availability of an acceptable quantity and quality of water for health, livelihoods, ecosystems and production, coupled with an acceptable level of water-related risks to people, environments and economies’ is more comprehensive and addresses both *provision* and *risk* perspectives.

The definition of risk can be expanded to accommodate the provision aspect, but this appears unhelpful as it both removes the constructive dialectic between risk and provision, as will be seen when specific aspects of domestic water and sanitation are considered, and also moves further from the use of the term *risk* in ordinary parlance.

**Figure 1.**
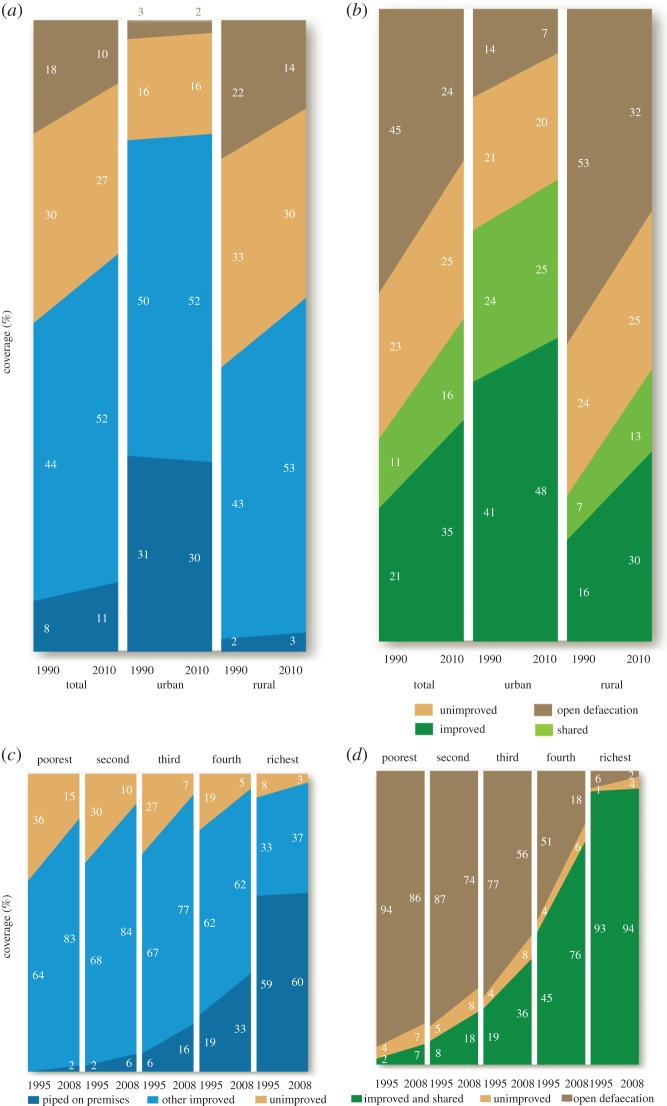
(*a*–*d*) Changing coverage of provision of various levels of improved water supply (*a*,*c*) and sanitation (*b*,*d*) between 1990 and 2010: for rural (*a*) and urban (*b*) populations of the least developed countries (LDCs; comparable data globally, for developing countries and for sub-Saharan Africa, are given in table 1); also for the population of three countries of South Asia (Bangladesh, India, Nepal, *c*,*d*) separated out by wealth quintiles. Both the actual percentage coverage and the rates of change in different wealth quintiles can be seen. Improved water has become more equitably provided, but both the coverage and rates of improvement for sanitation have diverged markedly. These are all from the JMP Report 2012 by kind permission of UNICEF and WHO [9].

There have been at least three phases in global water and sanitation development: the first, which the MDG period very much reflects, has been primarily one of *provision*. During the two decades from 1990, the number of people having improved water supply and sanitation in the world has increased by 51% and 68%, respectively (table 1 and figure 1). Now that coverage is so much greater [9], at 89% for water and 63% for sanitation, although the absolute numbers lacking water and sanitation remain globally huge, we can helpfully turn the second, current, phase in part to a *risk* approach in planning for the post-2015 period, under the classical epidemiological triad of time, place and person. What are the risks to provision over time as seen in terms of reliability and sustainability; what are the risks in terms of place, confronted by plans for WaSH in schools and health facilities; and what are the risks in terms of person? Who are the groups missing out, whether from poverty, ethnic discrimination, disability or age? So an emphasis on *risk* can sharpen the research needs and policy actions. However, with over 780 million lacking ‘improved’ water supply and 2500 million lacking basic sanitation the *provision* aspect of the Grey & Sadoff [8] definition remains critical, and we find this preferable to the later, more concise, definition in giving a balanced view of the global needs for domestic water and sanitation.

**Table 1.** Changes in numbers and proportions of people with improved water supply and improved sanitation between 1990 and 2010, by whether rural or urban, for the global population, developing countries, and countries of Africa South of the Sahara. The third numbers column gives the ratio derived by dividing the 2010 value by that for 1990. The final column for the numbers tables gives the percentage by which the 2010 value exceeds that for 1990; the final percentage coverage column gives the percentage by which the unserved percentage in 1990 has been reduced by 2010 (the MDG target for this was set at 50%. The table clearly shows that countries poorly served in 1990 may greatly miss the target in spite of a huge increase in the numbers served.

			number in millions: improved				percentage coverage: improved			
			1990	2010	ratio	%^a^	1990	2010	ratio	%^b^
global	water	urban	2142	3343	1.56	56	95	96	1.01	20
		rural	1896	2747	1.45	45	62	81	1.31	50
		total	4038	6090	1.51	51	76	89	1.17	54
	sanitation	urban	1720	2759	1.60	60	76	79	1.04	13
		rural	879	1603	1.82	82	29	47	1.62	25
		total	2599	4362	1.68	68	49	63	1.29	27
developing countries	water	urban	1346	2406	1.79	79	93	95	1.02	29
		rural	1586	2445	1.54	54	59	79	1.34	49
		total	2896	4840	1.67	67	70	86	1.23	53
	sanitation	urban	941	1849	1.96	96	65	73	1.12	23
		rural	565	1331	2.36	136	21	43	2.05	28
		total	1489	3152	2.12	112	36	56	1.56	31
sub-Saharan Africa	water	urban	120	263	2.19	119	83	83	1.00	0
		rural	134	264	1.98	98	36	49	1.36	20
		total	253	522	2.07	107	49	61	1.24	24
	sanitation	urban	62	136	2.19	119	43	43	1.00	0
		rural	71	124	1.76	76	19	23	1.21	5
		total	134	257	1.92	92	26	30	1.15	5

^a^Percentage increase by 2010 in number served compared with 1990.^b^Percentage reduction by 2010 in the % unserved in 1990.

The third phase overlaps the first two and is concerned with improving matters beyond the basic. To share a pit latrine with up to 29 others is not an adequate situation, nor is access to an ‘improved’ water supply necessarily safe or necessarily reasonably accessible. There is a wide acceptance of the need for a sort of ‘ladder’ of successive improvements such as water piped into the household and proper disposal or treatment of sewage [10,11], reaching to an eventual level where the improvements are a matter of conventional arrangements between users and utilities.

As the quarter-century for completion of the MDGs approaches in 2015, there is intense discussion of appropriate goals for the next quarter-century. This review aims to use the results and experience of the MDG period to illuminate this forward-looking debate, on the levels of domestic water security to be pursued and the scientific and technological progress required, in a water security context.

## 2. Millennium development goals and the growth of water targets and monitoring

Domestic water provision was one of the original targets of the MDGs, soon joined by sanitation [12], curiously cited as an ‘environmental’ target. The need to measure progress led to monitoring the provision of domestic water and sanitation by reliable methodology applied across the world. Publication of the resulting data and their use for advocacy has assisted progress. Indeed, as monitoring and measurement of what had been accomplished was taken seriously, a dynamic interaction between targets and indicators shaped the practical policies developed and it seems likely that future goals may have a similar formal structure. The goals, targets, indicators sequence tends to correspond to the sequence from political into professional into operations as political will is turned into technical and scientific action, whereas the upward flow of reliable data gives legitimacy to the process. Perversely, practical realities of monitoring, such as the adoption of an ‘improved sources’ indicator for drinking-water (because sufficient internationally comparable data on actual water safety were not available, and there was no feasible way to measure microbiological water quality in large household surveys, nor could actual water use be reliably measured on this scale) have impacted the targets and their meaning and implementation. There needs to be a continuous linkage between the different scales of work if encouragement and motivation are to work at all levels.

Monitoring results and targets are used for many purposes, including international comparison, policy development, planning, system and programme evaluation, benefit estimation and enforcement of regulatory compliance. Their content and nature are contested and equivocal, because different uses create different and sometimes conflicting demands. They have intended uses but are expected to serve other needs in a rapidly changing and unpredictable context.

### (a) International development targets for water sanitation and hygiene

The MDGs represent the most recent of several efforts to accelerate progress on W&S through international policy. The earliest of these addressed water and sanitation at a time when global population was four billion and predominantly rural. The 1980s focused on WaSH through the International Drinking-water Supply and Sanitation Decade. Unlike these earlier initiatives, the MDG WaSH target was pragmatic rather than inspirational. While its predecessors had targeted ‘water and sanitation for all’ or similar policy objectives, the Millennium Declaration formulated the target as ‘To halve, by the year 2015, the proportion of people who are unable to reach or to afford safe drinking water’ [12]—adopted because it represented a continuation of the proportionate decline in ‘unserved’ populations achieved in the preceding period. The international consensus around the policy importance of water and sanitation were confirmed through the announcement of the decade 2006–15 as the International Decade for Action, ‘water for life’ and of 2008 as the year of sanitation.

Whether the original MDG target represented an over- or underambitious target is open to debate. The increasing difficulty in delivering access to residual unserved populations, the need to keep up with ongoing population growth; the need to sustain an increasingly extensive and increasingly costly ‘stock’ of established services and infrastructures; and the inefficiency from redundant infrastructures associated with ‘upgrading’ from community sources to piped supplies might suggest it was ambitious. On the other hand, the increasing urban population serviced by organized utilities, improvements in governance, the benefits of human ingenuity in improving our understanding and implementation of engineering, policy, managerial and behavioural activities suggested that more rapid progress might be achieved. Whether through policy push or because of these underlying drivers, the water component of the MDG target was reached, based on the metrics of the time, in 2010 [9]. By contrast, sanitation—by which we mean the management of human excreta—did not appear in the first MDG formulations and was added in 2002 at the World Summit on Sustainable Development in Johannesburg in response to concerted advocacy. It was inserted by simple addition of basic sanitation to the already-modified water target wording and its achievability was not assessed. The widely different baselines for water and sanitation made the sanitation component far more challenging, because of the ‘halve the proportion of the unserved’ formulation; leading to unfortunate descriptions of sanitation as ‘lagging water’ and an unhelpful competition between the water and sanitation subsectors and subdisciplines.

### (b) International monitoring of water, sanitation and hygiene

International monitoring of status and trends in drinking-water and sanitation is provided by WHO and UNICEF through their ‘joint monitoring programme’ (JMP) [4]—itself a continuation of monitoring initiated in the 1960s [13,14]. While subject to fair criticism for inadequately responding to the ‘safe’ and ‘sustainable’ wording of the MDG target, their monitoring reflects one of the most internationally representative, consistent and comparable MDG monitoring initiatives.

The approach taken by JMP is to extract information from censuses and nationally representative household surveys on the source of water supply and means of sanitation reported by household members. Households are categorized as having or not having improved water or basic sanitation based on the associated technologies (piped supply or community well with handpump versus collection from a river; flush toilet or household latrine versus open defecation in fields, for example). These data are disaggregated by rural or urban setting, corrected to account for household size and used to estimate coverage levels. Coverage estimates from all available censuses and surveys are collated and a linear regression of coverage against time (year) used to estimate coverage levels and to extrapolate. Behind this simple approach, sets of rules constrain how far data may be extrapolated, accommodate national technology definitions that do not coincide with those used by JMP and so on. While this approach is largely taken for granted today, only in 2000 was the quantity of such data judged sufficient to adopt this approach, replacing questionnaire surveys of national government agencies. Advantages of the current approach include international consistency; data on actual water use from household members; and reduced cost by relying on large, multi-purpose, data gathering. Limitations include that householders are not a reliable source of information on safety and sustainability—which are therefore not accounted; that source data are point prevalences and fail to reflect the complexity of multiple sources, changing over time; all forms of service are considered equal despite their widely varying benefits; and there are few data from the smaller developing nations and from the more developed countries. Lessons to be learned from reflecting on international monitoring include sometimes rapid and substantive evolution in response to pressures and opportunities: for example, in the data shift above; coping with incomplete information; that despite the need for some ‘baseline’ situation, knowledge of that situation has been incomplete at the time of target adoption and has been retrospectively secured; and that indicators may serve a useful purpose even if they are an imprecise reflection of the situation.

Monitoring itself has important impacts on goals and targets as well as vice versa. Thus, the MDG ‘benchmarks’—the definitions of what constitutes ‘improved water’ and ‘basic sanitation’—match poorly to the MDG target wording (which incorporates notions of safety and sustainability), and were adopted because data were available. The interaction between goals and targets and their monitoring, and the evolution of monitoring alongside implementation are best recognized and interpreted rather than challenged and rejected. There needs to be enough continuity of methods to maintain comparability over time, but also the incorporation of new techniques as they become available for large-scale use, in parallel.

### (c) Progress according to joint monitoring programme/millennium developmentgoals indicators

Measured progress towards the W&S MDGs is summarized in table 1 [9]. Because the population of some countries has doubled in the past 25 years, the task of supplying water to additional people has often exceeded that of extending coverage of the original population, and information is expressed in several ways: increases in percentage coverage may be small even though more toilets may have been built than in the entire period to 1990 for example. Provision of toilets for the rich may vastly outstrip provision for the poor, and in some areas, notably India (but not Bangladesh), progress in the past 13 years has increased inequality (figure 1*d*), whereas improvements of domestic water supply have been more equitable, as in figure 1*c*, from WHO and UNICEF [9]. Extensive further data are provided in that publication down to country level. This wealth of material can be simply summarized: a great deal has been achieved, but the remaining task is huge. Moreover, ‘improved’ water and sanitation in many cases falls far short of reasonable expectation in that water may have to be carried to the household and may not be safe, and the ‘improved’ sanitation in figure 1*d* includes facilities shared between households that provide a lower level of access with potentially less hygienic facilities and lesser physical security for users, especially where facilities concerned are public.

The period up to 2015 is characterized by an attempt to increase the construction of more rural water sources, particularly from groundwater, and to keep up coverage for increasing urban populations by a mixture of in-household piped water and standpipes. Sanitation attention has focused on provision and use of toilets at household level. By contrast, issues of reliability, continuous availability, provision to the poor, particularly of sanitation, water safety, systems for dealing with excreta beyond the household and equity, have been neglected. The way in which the difference in coverage with WaSH between 1990 and 2010 is viewed depends critically upon presentation, but there has been an increase in the number of people with access to water or sanitation facilities in the world of 57% (82% for developing countries). The problem of maintenance of facilities is therefore of much greater importance than in 1990 (§4*d*). Problems and solutions for the water delivery process are examined by Hope & Rouse [15].

Available information suggests that there are large and often consistent inequalities experienced by different segments of society. Everywhere, use of ‘improved sources’ of drinking-water is substantively lower in rural than urban populations (figure 1*a*). Globally, 81% of the rural population has such access, whereas the urban figure is 96%; in sub-Saharan Africa, these figures are 49% and 83%, respectively (JMP) [9]. While a human rights perspective would suggest that such a pervasive inequality would merit remedy, debate often suggests higher service levels or standards for urban than rural areas. There are similar inequalities across wealth quintiles, most markedly for sanitation in South Asia where in 2008 only 6% of the richest quintile had unimproved or no sanitation, whereas this was the lot of 86% of the poorest quintile (figure 1*d*), and between genders where, for example, the burden of physical collection of water is carried disproportionately by women and girls [16].

Even where water scarcity is widespread constraints to access are inequitably distributed, whether through differential distribution (protection of supplies to wealthier districts); differential adaptive capacity (wealthier households constructing larger or lower level water storages in order to secure and store water) or differential experience of scarcity (exacerbation of the gendered water collection burden).

### (d) Critique of the millennium development goals implementation and joint monitoring programme monitoring experiences

While the MDG drinking-water target was worded in global terms, it has been widely applied on a country-by-country basis. Maps and tables depicting countries as ‘on’ or ‘off’ track have been generated and have informed policy-making. The ‘halve the proportion of the un-served’ formulation is such that, applied at a country-by-country (or other subglobal scale), greatest demands for progress are made of areas where baseline conditions were poorest. In human rights terms such a demand makes a positive contribution towards increasing equity. However, it also creates the counterintuitive situation where those countries which have made most progress may also be ‘off-track’ (table 1).

The ‘reduce by half the proportion’ wording reflects an astute handling of population growth without being explicit about it: for most developing countries, far more water and sanitation facilities had to be established to cope with increasing population than to reduce the gap in provision for the initial population. However, in doing so, it failed to recognize the success of efforts where achievements were greatest.

The current WaSH goals and targets are least satisfactory in two areas: neglect of upstream sustainability aspects of water, for example reliability in a water source whether reduced by climate change or human intervention; see §3*d*), and of downstream aspects of sanitation (where faeces from inadequate sewage disposal contaminate the human environment), where progress is patchy and not always thought through. In both these situations, a risk approach could usefully be systematically applied, especially as these topics both impinge on the ‘big water’ issues (§4*f*).

Some remaining monitoring problems are statistical, such as the instability of the baseline, which depends on linear regression of coverage over time, because the actual 1990 data were inadequate. Others follow the lack of water quality or actual water use measures within the survey methodology adopted for its other advantages. Yet others are a consequence of necessarily simple categorization for global use: some types of well may often yield safe water in rural areas but less reliably in cities; some types of sanitation facility sharing between households may be satisfactory and others not. Most generally, the basic facilities that are a great advance on zero provision are still far from ideal and higher aims are to be sought, so that their relative adequacy should be accommodated in the international monitoring and national planning. Simplicity, required for political motivation and achieved through the binary classification, is soon lost in efforts to describe this complex situation. Moreover, global comparability, achieved with much effort, limits utility of the data at national level and below for planning. These difficulties must be addressed by any post-2015 goals and targets. However, the baseline for the next quarter-century is very different from that of 1990 largely as a consequence of efforts in the MDG period.

## 3. Planning for post-millennium development goals 2015

There are three major differences between the pre- and post-2015 periods. The substantive developments in monitoring have been described in the preceding section and are analysed here. In addition, post-2015, the baseline situation is vastly better as a result of the MDG push; and a ‘human right to water and sanitation’ is now recognized, providing normative specificity on dimensions and levels of adequacy. A further advance in recognizing the importance of hygiene behaviour is yet to be achieved.

### (a) The implications of developments in monitoring

The main monitoring achievements since 2000 have been independence (perceived and real), comparability between countries, well-defined variables and transparency of process. These have been achieved partly by, and partly at the expense of, some distance between global and national monitoring. These two aspects could usefully be brought closer together, partly for efficiency—so that they are mutually supportive—and partly because more thorough and detailed monitoring will require greater resources that will not be forthcoming unless they can serve both needs. This can be achieved by making the monitoring informative to more audiences, and particularly to national water programmes and utilities. It can also be a catalyst for building up national water regulators and the national water monitoring systems that will take over from the UN organizations in due course as sources of information for international monitoring.

Both for continuity as a baseline and as a ‘gold standard’ within countries, monitoring over the 2015 period will therefore likely keep nationally representative household surveys as a core data source. However, they will require supplementation in order to add value and justify their collection. Supplementation is likely to involve new or amended indicators to reflect water safety, equity including minority groups, a ladder approach to service provision in and beyond households (schools, healthcare settings and so on), a more comprehensive view of sanitation). Supplementation would also usefully include geo-referencing to enable greater resolution in analysis by disaggregating data into the settlement types of table 2.

**Table 2.** A possible classification of populated areas primarily on a residential population density and geographical basis. If monitoring data are disaggregated on this basis it will make them more meaningful to provider organizations, will tend to separate different types of provision technology and problems, and will facilitate international research categories.

urban	1	capital city (excl. 3,4.)
	2	big cities (excl. 3,4.)
	3	centre urban slums
	4	peri-urban slums
	5	towns
rural	6	settlements
	7	dispersed
	8	specialized/other

### (b) The changed baseline situation for 2015

At the time of formulation of previous international development targets, the situation was dominated by insufficient provision. Formulation of goals and targets for the post-2015 period confronts a fundamentally different baseline situation: the need to extend coverage is accompanied by the need to maintain accumulated infrastructures and services, reflected in the demand to monitor ‘sustainability’, and the demand for higher levels of service—providing greater social wellbeing and economic benefits—related to the demands not only to improve matters in aggregate but also to increase equity.

After half a century of international WaSH monitoring, there has been minimal change in indicators (household use of technology types) and reporting (proportion of population living in households with more desirable technology types). Given accumulated progress and the driver of a human rights perspective (§3*c*), it is unlikely that long-established indicators will remain adequate The political, economic and technical risks associated with such change have not previously been tackled. *Political risk* is associated with, for example, the mixed message of ‘success—MDG target on water reached’ accompanied by demands for policy support to do more include implicit recognition of the inadequacy of what has been targeted, accelerated and achieved. *Economic risk* because the shift in focus from community to household water and from a user-only to system-wide perspective on sanitation together increase costs substantively. *Technical risk* because the institutions, human resources and management systems attuned to extending provision of basic services may be poorly aligned with the needs of maintaining established systems and enhancing service levels. These risks broadly reflect the augmentation of a provision- with a risk-based stance. Certainly, at a national level and below, water service will need to be emphasized strongly, and is supported by a risk approach. A risk inherent in the provision perspective is that sustainability (in the sense of continued reliable functioning of facilities; §3*d*) may be inadequately considered and that in consequence the impacts of efforts will be undermined through premature failure, which is widely documented. A ‘risk’ perspective may complement that of provision in capturing this.

### (c) Consequences of human rights approach

The recognition by the United Nations of a ‘human right to water and sanitation’ [7,17] has in one respect greatly simplified statements of the goal for domestic water and sanitation provision which must surely be 100% coverage. In other ways, it has complicated the conceptual framework as there is much diversity in understanding of the ‘right to water’, the concept of rights to goods and services is philosophically less than secure [18] even though politically and legally valuable, and it provides high-level leverage on heads of state and governments.

The most important practical value is that this status requires that there be no discrimination against particular categories of people. In consequence, governments have accepted the need to emphasize provision for the poor and marginalized and so increase equality. The implications are substantive: for policy and implementation where an explicit targeting of vulnerable and disadvantaged groups clashes with simplistic cost–benefit perspectives and maximizing coverage increases at minimal cost; and for monitoring, because current approaches are inadequate to assess specific discrimination—although data analysis can enable the assessment of equity trends.

The concept of ‘progressive realization’ enables water and sanitation to be viewed as a process rather than viewed an all-or-nothing state. Its importance includes the extension of international development target relevance to all countries and populations as inequity and risks remain in the developed nations. By placing all countries on a continuum of adequacy/risk, it raises the challenge of comparing countries with widely varying achievements; however, it is of limited value in sequencing resource allocation.

This human right broadens responsibility for provision: when household coverage was the yardstick of progress, the state, utilities and donors were the primary audience. If rights include, for example, disabled persons and water and sanitation in schools and workplaces, then operational duty bearers include many more people. Indeed, a focus on operational *responsibilities* to accompany that on *rights* may sharpen focus. The human right to water and sanitation strongly implies a need to think of risk to individuals and beyond a risk tolerable to society. Many of these issues have not yet been adequately thought through.

The dialectic resulting from the provision/risk modes of water security provides a constructive way to improve the mechanics of *progressive realization*, the way in which human rights discourse handles priority setting, at the country level. Proposals for post-2015 targets identify ‘basic’ levels for both domestic water and sanitation, with their indicators and currently well-tried methodology. They also describe ‘intermediate’, rather better situations in which, for example, microbiological indicators are used for water safety and sharing is reduced for sanitation. The primary target is to provide the basic minimum for all. The study of risks, whether of quality or access, then identifies issues needing attention either individually, or by combining attention to several issues in revised target levels of provision for widespread application. This alternation between the analytic and normative approach may also be useful as separating out what is primarily of national and global interest, respectively, so far as reporting is concerned. In addition, because basic levels are likely to be of universal application, whereas higher levels are more likely to differ in content depending on ecological context, and also to become of less concern to external funding agencies, there will be increasing room for diversity as the overall level of W&S availability improves.

### (d) A water security perspective of the current phase

The overall goal is now driven particularly by agreements on the human right to water and sanitation and responsibilities of countries in consequence.

A *provision* perspective, alongside human rights perspectives, highlights need to secure universal use of basic water and sanitation and simplifies the primary goal to 100% coverage. Statistically, this looks achievable by 2040, using metrics applied to date. Nevertheless, there is then much room for defining the nature of these basic supplies that are a human right. Incorporation of, for example, a water safety component would substantively depress estimates of status and trends. This basic level will not be satisfactory to large parts of the world's population.

Complementing *provision* with a *risk* perspective makes it evident that basic provision does not achieve an acceptable level of risk. These risks can be viewed under the traditional triad of time, place and person.

The *risks* to provision over *time* can be seen in terms of *reliability* in the short term and *sustainability* of WaSH and associated consequences for health and development (§4*d*). They are reflected in the MDG target formulation through reference to ‘sustainability’ which has not been accounted in monitoring to date. Doing so is complex, in part, because the word conveys different meanings for different constituencies, including whether a service is maintained over time, whether the demands it creates are economically, socio-culturally and environmentally ‘sustainable’; and whether these two can be sustained in the long term (inter-generational equity). Sustainability interacts with broader aspects of water security (§4*f*).

There are practical difficulties inherent in measuring sustainability, for instance defining minimum acceptable infrastructure lifetime and a ‘reasonable’ level of failure. Reliability and frequency of supply have become more critical with increasing population. Some South Asian supplies are only available on 2 days each month, and many piped supplies are for a few hours daily. The combination of social, managerial, engineering and political factors that determine the acceptability of this is inadequately understood, as are the corresponding inter-disciplinary and inter-sectoral approaches required to rectify it. Although clarifying, monitoring and implementing sustainability will require much more work, improving reliability is a simpler and urgent task.

*Risk* associated with the notion of *place* leads to recognition that individuals use both water and sanitation outside the household which has been the principal focus of provision and the exclusive focus of international monitoring to date. The majority of a population will use WaSH services in schools and workplaces for many years. Many will experience them in special risk settings such as healthcare and some when they are reliant on service provision by some external agency as refugees, internally displaced populations or members of the prison population. This wider perspective will reduce major exposures to unsafe water use and have huge educational potential. It is in primary schools that hygiene behaviour (such as washing hands with soap) is learned, and the standards of school domestic water and sanitation will be formative.

What are the *risks* in terms of *person*? Who are the groups missing out, whether from poverty, ethnic discrimination, disability or age? As basic household provision approaches saturation, countries at all levels of development are likely to be challenged in reaching some subpopulations. Monitoring of provision to them has special challenges as they may disappear in aggregate statistics. Approaches require either specifically targeting identified subpopulations or reliance on larger-scale indicators of increasing equity (rural–urban, across wealth quintiles). There is much value in international cooperation to enhance understanding of how to tackle such intractable situations where established mainstream approaches have proved inadequate.

A risk approach applied in developing-country situations may lead in several directions: in communities with *basic* water supplies a risk approach may, for example, indicate problems of limited access and imperfect water quality. Action to improve these may be piecemeal attention to the specifics, or it may lead to comprehensive review, revised target specifications and return to *provider*mode for better provision or upgrading of WaSH facilities. In other words, when there are multiple risks, a solution may be the major reconfiguration of what is either provided, or to be provided, as an improvement on the *basic* situation. This is the way in which *intermediate* water and sanitation provision and potentially a ladder approach [11] arises within a water security framework.

### (e) Water security and the next quarter-century

What are the implications of a water security goal or framework for WaSH over the next planning period Can it be more than a slogan or label, and help provide an intellectual framework that will support equitable and sustainable WaSH and the necessary research to support this? The general case has been explored above and applied to the current situation as the MDG period draws towards its close.

When the circumstances around the past 25 years of progress towards WaSH targets are compared with, and used to enlighten, the plans for the next quarter-century, issues of water security and of risk emerge as a way to gain focus. In looking at the forthcoming period, as is being done in anticipation of the 2015 end year for the MDGs, several high-level questions about goals and targets arise, with multiple problems of measuring progress.

The household focus of the MDGs has led to underemphasis on aspects of WaSH beyond the understanding of individual householders, ‘upstream’ water and ‘downstream’ sanitation particularly. While the proportion of total available water that is required for WaSH is in aggregate small, there are many places where local water scarcity is the dominant concern and where boreholes deliver erratically or yield water that is unsafe. A risk approach will lead to a more systematic analysis of groundwater adequacy.

A conceptual framework and policy around water security (provision and risk) rather than household coverage would arguably have picked up the relative neglect of water and sanitation in schools more readily than has happened. Similarly, such a policy will point towards a balance between maintenance of what is there and increasing household coverage, whereas household provision as a target tends to prioritize construction above maintenance.

In a goal-orientated system, important issues which failed to reach the final targets or indicator formulations, or which cannot be reliably measured at reasonable cost are neglected. There is also a practical limit to the number of variables that can usefully be aggregated to global level, but far more that are required for national and local purposes. The risk/provision of a water security framework is useful at both scales and promotes a critical analytic approach. When evaluating impacts (such as those on health) alongside outcomes, risk analysis of the gap is an educational as well as analytic tool to ensure that attention is given to behavioural and cultural inputs. The chains of events linking inputs to impacts may be long, but informative [19].

## 4. Water security and key emerging problems in water sanitationand hygiene

Here, we review several issues of major concern in post-2015 planning and examine whether water security, in conjunction with the right to water and sanitation, can contribute usefully. Key points of debate include indicators of water quality, improving the balance between construction and maintenance of the now-extensive water and sanitation infrastructure, priorities in reducing low-level versus intermittent organic pollution, innovations in sanitation, the institutional consequences of a water security approach and the relation of WaSH to broader aspects of water and wastewater management.

### (a) Water quality measurement and delivery: coliforms, household water treatment and epidemiological risk

There is extensive evidence for the adverse impact of polluted water on human health. Water safety is fundamentally a measurement of risk, whether of infectious diarrhoea, schistosomiasis or arsenic poisoning. The limitations of ‘improved water source’ as a proxy for safe water are outlined above.

Water collected from some improved sources is unsafe owing to contamination with pathogens or toxic chemicals and through inadequate sanitary protection. Adjusting estimates of water trends to account for this would place the water component of the MDG target badly off-track [20]. Even water manually collected from ‘improved sources’ and stored is often re-contaminated. While literature review on balance points to such contamination as a health hazard, more epidemiological analysis is required. Moreover, the prevalence and degree of contamination of water between collection and use vary widely between and within countries [21]. These factors suggest that future monitoring must include some direct assessment of water safety.

Anticipation of new targets has already spurred development of cheaper methods for testing of *Escherichia coli* and some chemicals in water. Further developments of relevance to both developed and developing nations may include simplified low-cost testing for faecal contamination that is not reliant on specialized expertise, and automated testing—reporting able to inform decision-making in appropriate time frames for public health needs.

Water safety provides an example of a situation in which much and increasing subnational monitoring is already undertaken by utilities, regulators and entities supporting rural water programmes. This suggests opportunities in incorporating data from multiple sources. Indeed, such data may provide opportunities for rapid developments—in the same way that data from nationally representative surveys rapidly supplanted that from governmental questionnaires. In future, this may include data from smart metering of water use and the technical innovations in coming decades.

Rather than see international development targets as a spur to externally driven testing, it might preferably be seen as a domain in which progressive development of national capacities may spur synergistic linkages between national systems and international monitoring and future international WaSH monitoring that is driven by effective national systems in countries worldwide. Interim measures will be essential to bridge the divide between the current situation and the desired future of effective, integrated and representative national systems.

The drive to provide ‘improved’ water supplies has been largely successful in urban situations, despite population growth and even in impoverished cities. Future goals and targets for urban areas may therefore increasingly focus on provision of water piped to the household or compound. Large populations now lie in a transitional condition between the pre-basic situation where water is carried from unimproved sources and people are continuously exposed to unsafe water and the fully developed situation where there is reliable safe water to drink in most of the places where one might be.

Such populations are intermittently exposed to source failure or supply interruptions and unsafe water and, in consequence, are also transitional from endemic high-level transmission of faecal–oral infections (chiefly the diarrhoeal diseases) to a situation where they are rarely exposed to these infections. For the water-based diseases such as schistosomiasis, the environmental control strategy is clear: to keep out of infective waters, because the risk is high. It becomes a policy of avoiding the residual exposure to risk of infection.

In matters of water access and *quantity* used, there is a progressive increase in health benefits as use rises, particularly from the lowest levels, and they tend to level off once water is readily available. The situation over water *quality* differs for infections, in that the key variable is not how much microbiologically safe water is consumed, but how much polluted water continues to be ingested. Risk analysis therefore needs to be concerned with sources of unsafe water, often from outside the home.

Recent studies have used quantitative microbial risk assessment [22] to show that the occasional consumption of polluted water is highly dangerous to health. This involves modelling approaches that make assumptions about the infective process, but some studies keep relatively close to data [23,24]. Epidemiologically desirable information can be hard to gather, especially for uncommon events that are difficult to observe because of ethical necessity to intervene. However, an emphasis on water security and risk points to the need for data on these events. In particular, research questions include whether to use scarce resources to improve the microbial quality of relatively good sources, to make great efforts with household water treatment to both improve the water above source quality and deal with low-level re-contamination of the main household water supplies, or to put that effort and resource into trying to stop consumption of unsafe water outside the household.

### (b) Sanitation

Politically, domestic water provision has more appeal than sanitation, and components of sanitation that take place within the household are given precedence over the management of sewage and excreta downstream of the household, which has all the problems of a common good. These two facets compare with provision and risk perspectives, respectively. It suffers neglect, and falls between the sanitation agenda narrowly conceived and environmental management activities. Where utilities provide sewered services, it falls within their responsibility, but treatment of the collected sewage is uncommon in upper-middle countries where sewerage coverage is relatively high [25]. Where on-site pit latrines and septic tanks are involved, their contents may reach watercourses untreated because of improper disposal after emptying, system failure (e.g. of leach fields) or driven by factors such as flooding potentially exacerbated by climate change. In sanitation, provision is the dominant need (table 1), but there remain doubts over what form of provision can be extended to effect in the cities.

It can be argued that in water and sanitation beneficial use of wastewater and excreta is the great scientific, technological and environmental challenge or opportunity of the coming quarter-century and is of special relevance to poor rapidly developing countries. There are doubts about the economic feasibility of classical sewerage and about its logic: to dilute excreta with precious water and then separate the two again is costly and energy-intensive. Ongoing experiments with dry or semi-wet sanitation will benefit from sophisticated microbiology but the challenge will be to go to scale at affordable cost and without making excessive demands upon users. In some developing countries, rapidly expanding urban peripheral areas include urban farming and market gardening using wastewater that may or may not have been treated. While there is clear evidence of communicable disease hazards for some workers and consumers [17], a full risk analysis of trade-offs may lead to better balancing of risks. The conservatism of utilities in this area is understandable especially in rich well-watered countries where the systems are well-established, but greater innovation may well benefit countries with water scarcity, and much greater scientific input is required.

The value of sewage for irrigation is in terms of its water and plant nutrient content, particularly nitrogen and potassium (reducing eutrophication of freshwaters that would otherwise have received the effluent and fertilizer runoff), but industrial wastewater may contain high levels of toxic substances needing source-exclusion, removal or detoxification, a field for further research.

The priority of *downstream* sanitation interventions is controversial; the two predominant but conflicting views are that the urgency of intra-household toilet provision overrides the need for full excreta management; or the lack of attention to excreta once it has left the household constitutes a major environmental and health problem. The former position rather neglects the fate of, for example, latrine pit contents, whereas the latter ignores the usual operational response which is to build treatment facilities that are costly to build and operate, rather than to improve transport and disposal of faecal sludge. Priorities and solutions are dependent upon residential population density, degree of industrialization and the options for faecal sludge disposal. The downstream risks encompass both health risks to neighbours and damage to the environment. Both require that faecal sludge be safely removed from the area.

A comprehensive risk approach to the downstream aspects of sanitation after excreta leave the household has yet to be worked through and risk analysis has largely tackled specific questions at a single stage of one sector, and the overall strategy on a systems basis is less clear. It will need to involve other domains of water.

While sanitation as part of WaSH clearly fits at present within water security, it is reasonable to ask whether, if excreta disposal becomes more separated from water for flushing, it will logically remain part of a water security agenda. If history is any guide, then this will not happen rapidly or completely in most countries. There will, in any case, be reasons to stay with waterborne sewage in several situations: where water is not scarce, and the sewers have already been laid; where strong religious or cultural reasons for copious water use remain; where irrigation water is required and the geography favours waterborne transport of nightsoil as a manure, and in urban areas where waste water needs to be removed from crowded areas.

If urban farming is to become widespread in developing country cities to cope with wastewater and runoff, then it is likely that excreta will remain part of the picture as well. To the extent that excreta are treated dry and on site they could be thought of as a less integral part of water security. However, this appears a somewhat abstract approach: water security will depend on that alternative system being available and functional; to reach the situation where no excreta or their derivatives reach water bodies seems utopian, at any rate for other than the richest countries.

Sanitation requires more thorough analysis in relation to both water security and human rights. The need for provision of basic sanitation is evident on grounds of health, human dignity, safety and environmental management of the most elementary kind.

### (c) Water security and the operation and maintenance aspects of water sanitationand hygiene

The area that in formal terms lies between *provision* and *risk* perspectives concerns rehabilitation, maintenance and aspects of operation. Failures in maintenance are a perennial theme for public utilities in most developing countries and construction of new facilities has diverted skilled staff away from maintenance. Foreign financial aid has been more readily available for new work than for maintenance. A helpful consequence of a risk approach, if implemented systematically, is that it should change the approach to unreliable and ageing infrastructure towards prophylactic action and scheduled maintenance and renovation. A risk approach also encourages a systems view of the issues, so that hygiene behaviour forms a natural part of the analysis of problems.

In monitoring, maintenance failures are reflected in continuity of service; where discontinuity occurs at three different scales—managerial: planned and predictable supply for example on an hours per day and/or days per week basis; seasonal discontinuity where reliability declines in response to seasonal variation in either supply or demand; and breakdown discontinuity of widely varying duration. Implications of a water security approach for WaSH monitoring are substantial in the medium and long term, but for global monitoring and as a ‘gold standard’ reference the current survey and census data will remain essential. The needs at national level (and they will be, in practice, used internationally for funding purposes) will increase under any scenario, water security or not, but particularly when a risk view is taken of maintenance. While anecdotally the countryside of many least developed countries is littered with derelict wind pumps and storage tanks, dry small reservoirs and broken handpumps, longitudinal and indeed rigorous data on facilities and their functionality are scarce and from users are lacking, although much required.

There are good reasons for the scarcity of longitudinal data from users (although the in-depth sites in Africa provide a demographic basis for over 20 sites, for example) but they are required at research level to provide an understanding of the dynamics of supply in poorly served countries as a basis for policy formulation, and at an operational level data are required on the infrastructure. Electronic methods have increasing potential for monitoring water delivery infrastructure, but the monitoring of basic sanitation is presently less tractable.

### (d) Institutional issues

A water security perspective, when combined with other changing aspects of WaSH, has implications for the way monitoring is structured, the way in which primary data are categorized and communicated, and the way in which these inform implementing agencies, so as to maximize provision and diminish risks. It has substantial consequences for capacity building. A risk approach makes analytical demands on national professional staff. Hopefully, it will increase the status of maintenance plans as a skilled task, and introduce more of a systems approach to WaSH. It will certainly both make demands on and require development of professional and research capacity at country level and should raise the status of those involved.

The conventional utility model of *water supply* can probably reach far down into the poorest households in urban situations because of the dense populations in slums and correspondingly short extra pipes required; because evidence suggests that such households often pay more, in practice, than those in wealthier and better-served neighbourhoods; moreover, reduced transaction costs and possible use of smart metering tend to bring the urban poor into the range of households that can be supplied by utilities. The problems are greater in rural areas. By contrast, the utility *sewer* approach to excreta in cities may break down at a higher income level and community-based cooperative sanitation organizations have been successful in diverse urban areas.

The risk perspective combined with the extent of infrastructure in place will change the balance towards maintenance from new building. Communication of monitoring results will need to be more widespread—to politicians communities and professionals. For sustainable provision, one will look to utilities that work at scale and tend to see ‘customers’, but these same utilities tend to overlook the poor and marginalized risk groups. Those are often served by NGOs and self-help groups that perceive households and communities rather than customers. Monitoring findings need to be communicated to both types of providers, with their differing perceptions, and should bring them closer together, because both have essential inputs for equitable provision of improved water and sanitation.

### (e) Disaggregation of monitoring results for communication

The way in which data are presented to some extent creates the resulting discussion. Hence, there is much legitimate pressure to disaggregate monitoring data by income/poverty level, by ethnicity, by gender, etc. These are all steps towards equity but speak primarily to monitoring groups (rightly) rather than to users or providers. This section, by suggesting disaggregation by population density and geographical area, aims to interest utilities and NGOs in the data and also to facilitate international interaction for technologies and research on better provision between those working in comparable environments internationally.

If global monitoring data are also to mesh with national and subnational data and particularly to be of relevance to providers, then they need to be more extensive. Moreover, as the post-MDG period openly focuses upon the underserved, there is a need for data disaggregation—to sharpen understanding of where the problems lie and to bring the datasets closer to the providers of water and sanitation services. What should be the primary disaggregation categories of national data? It is already clear from the JMP's work that wealth quintiles are highly informative and are key indicators of inequality of provision. So too are data on the other categories of those underserved or discriminated against. But they do not, on their own, speak to utilities and other providers. If the data can also be geo-referenced and tabulated by residential population density (table 2), then comparison across countries is fairer and more meaningful; stratifying delivery problems into categories which have commonalities across many countries becomes possible; and data are more related to the areas of responsibility of utilities in countries.

The provision made, its economic basis and technology, will differ between rural dispersed populations and villages. Urban needs differ between large cities and small towns, and between the inhabitants of inner-city slums and those in poor peri-urban areas. On this basis of population density, which is now becoming detectable by remote sensing, a suggested functional classification of areas is given in table 2. Certain people will have needs requiring special provision, such as nomadic herders in deserts. Because there may be more similarity between slums in different countries than between richer and poorer city dwellers in the same country, the proposed disaggregation between places brings research problems and risk categories closer together and is conducive to regional and global research planning. The data are categorized in a way congruent with patterns of provision, and we consider that population density is the primary subdivision of data that best points to the type of remedial action required, which may include new management models for rural services.

We suggest that in future monitoring should be aimed at providers as well as users, and that further provision of services can be usefully combined with a risk perspective. The poor and other deprived groups may be seen as falling into two types: those deprived people who live aggregated in geographically definable areas and those dispersed among better-served people. Water and sanitation services are geographically delimited. Provision for the dispersed unserved, poverty-stricken urban households scattered among the better-off can perhaps best be ensured by allocation of defined areas to utilities who assume responsibility for providing services to all those living within the area, although experienced utility managers naturally prefer subsidy of water bills to be provided by government. The areas of aggregated deprived people can be the subject of specific programmes.

### (f) Water security, integration of water management and ‘one water’

The preceding sections have investigated whether water security provides a useful conceptual framework for progress in WaSH and how WaSH might fit into a broad water security global goal. If the latter should develop [6], there are several interactions between WaSH and other domains of water which may be facilitated, both ‘upstream’ and ‘downstream’ of domestic water use. These other domains have collectively sometimes been called ‘big water’. Conventionally, the term has been used for water resources, issues of the global water cycle and measurements of large uses or masses of water at the global level and also the allocation of water in international transboundary large river catchments. However, the other aspect of ‘big water’ is made up from the hugely replicated activities of individual households and their specific needs for, and uses of, water, as is the case with WaSH.

WaSH is one of several users of water resources and, with suitable treatment, waste water can be returned to the resource pool. ‘Big water’ issues are addressed elsewhere in this volume and most daily WaSH activities are relatively separated from overall water management in both inner cities and truly rural areas, but that is less so in one situation.

Urbanization, proceeding rapidly in countries with low income, creates large peri-urban slum areas, often on marginal land—hilly, or prone to flooding, complicated by lying beyond the edge of the city boundaries in many cases, where water supply, sanitation, wastewater and shelter quality are all problematic and where each impinges on the others. Even though different agencies may deal with these problems operationally, it will be necessary to view them together as ‘one water’ (by analogy with one medicine/one health) in overall planning. This overlaps with contemporary discussions using the terminology of ‘cities of the future’.

‘Upstream’ combining water provision for domestic use with that for productive uses has been thoroughly discussed recently, but the economics of water quality improvement, in ways unnecessary for agriculture, complicates this, and the varied water needs of different people in the same community are among the practical impediments to this as a general approach.

WaSH may need to give a higher priority than hitherto to water resources management, especially in relation to ground water. This will require clear understanding of the value (benefits) of water in its diverse uses in different settings. The reliability, sustainable yield and seasonal yield variation of tube-wells in African rural areas need to be better understood. Hydrological risk requires more attention in relation to WaSH.

Much current WaSH literature is concerned with notions of ‘scalability’ and ‘sustainability’. These are issues closely related to provision and risk, respectively. Scalability is concerned with how robust an upward change in provision will be: the transition from research to operations. The criteria for needing innovation, the robustness of what it achieves, and the assessment of its operational limits in terms of both place and people are all best approached from a risk perspective. Much current thinking concerning scalability, as with discussion about risk, concerns the specific circumstances of population groups in their settings. With sanitation, as with water provision, the population density of settlement is crucial, as the problems of scaling up solutions in sparsely populated areas of rural areas are very different from those in high-density urban slums where sanitation upscaling involves interactive problems with drainage, solid waste management and transport.

Sustainability has for several decades been considered in environmental, economic and social terms. All three have contemporary relevance in the context of WaSH. *Environmental* sustainability because of concerns for the sufficiency of the underlying water resource base and the consequences of excreta disposal and wastewater for ecosystems; *economic*, for concerns about the ability of populations in both the developed and developing nations to financially support the services being provided—including concerns for equity and access for all; *social*, reflecting, in part, the emerging concept of an ‘enabling environment’ of interacting factors at household community and national/state levels that will tend to facilitate or obstruct the continued effective delivery and use of desired services. Environmental and economic aspects of sustainability require negotiation with water resources management and agricultural water users, respectively.

Transaction costs of work involving several domains of water security are not trivial. Experience with integrated water management in recent decades has sometimes been very time-intensive but with limited output. The optimal degree of cross-domain collaboration needs to be assessed. The extent of complexity and relevance of water and sanitation to diverse domains is illustrated by the fact that the number of UN entities engaged in water issues now exceeds 30.

## 5. Conclusion

The broad water security definition, combined with the human right to water and sanitation, has the potential to provide a sound policy base for sustainable improvement of the human condition in a fragile environment. Much detailed exploration of the implications of this approach is required, and its implications for the way targets are set and monitored. It can support a better view of the balance between new construction and maintenance than has been the case in recent decades, and foster a better approach to downstream sanitation in which risks are more carefully examined. It can point towards more longitudinal monitoring and sustainable water and sanitation systems at all stages of economic development, but only if used as a driver of thorough work and not merely as a new slogan or fashionable phrase.

The combination of a water security and a human rights approach will not be easy but is essential. A difficulty relates to the tension between human rights-driven priority to increase equality and the economically driven priorities that emerge from simple cost–benefit considerations. The strength of human rights lies in its concern for universality and equality, and its weakness in relation to goods and services is its limited utility in setting priorities and quantification. But these are the great strengths of a risk analysis within water security. The concept of ‘progressive realization’ also provides a spur to continual improvement and a lens through which to compare progress in countries at different levels of WaSH attainment. The hard face of human rights is the effective allocation of responsibility (duty bearing) at operational level.

This review aimed to address the area of water security as a possible ‘umbrella term’ under which WaSH could fit, along with other aspects of water, in a framework of future international development goals. Such a framework would inherit the strengths of the MDGs—including a framework of clear targets and indicators. Our analysis shows that a water security lens may provide systematic benefit if informed by a balance between provision and risk perspectives, enlightened by human rights insights. In combination, these can assist in defining the problems and pointing towards necessary actions. Actions can, in turn, be grouped together as under provision mode and service quality improvement related to a progressive realization; and a risk mode providing a tangible focus for the sometimes abstract or imprecise concept of sustainability. Both provision and risk thereby inform policy and programme development and are informed and supported by a dynamic approach to monitoring. Such a conceptual framework may provide a balanced approach helping to improving water and sanitation for the world's people over the coming quarter-century.
